# Women’s knowledge and perception of male circumcision before and after its roll-out in the South African township of Orange Farm from community-based cross-sectional surveys

**DOI:** 10.1371/journal.pone.0173595

**Published:** 2017-03-24

**Authors:** Barbara Maraux, Pascale Lissouba, Reathe Rain-Taljaard, Dirk Taljaard, Julie Bouscaillou, David Lewis, Adrian Puren, Bertran Auvert

**Affiliations:** 1 INSERM U1018, Villejuif, France; 2 Médecins sans Frontières, Paris, France; 3 Progressus Research and Development, Johannesburg, South Africa; 4 CHAPS, Parktown, Johannesburg, South Africa; 5 Médecins du Monde, Paris, France; 6 Western Sydney Sexual Health Centre, Parramatta, Australia; 7 Marie Bashir Institute for Infectious Diseases and Biosecurity and Sydney Medical School-Westmead, University of Sydney, Sydney, Australia; 8 Centre for HIV and STIs, National Institute for Communicable Diseases, National Health Laboratory Service, Johannesburg, South Africa; 9 University of the Witwatersrand, Faculty of Health Sciences, Johannesburg, South Africa; 10 AP-HP, Hôpital Ambroise Paré, Boulogne, France; 11 University of Versailles-Saint Quentin, Versailles, France; University of Pittsburgh, UNITED STATES

## Abstract

The roll-out of medical male circumcision (MC) is progressing in Southern and Eastern Africa. Little is known about the effect of this roll-out on women. The objective of this study was to assess the knowledge and perceptions of women regarding MC in a setting before and after the roll-out. This study was conducted in the South African township of Orange Farm where MC prevalence among men increased from 17% to 53% in the period 2008–2010. Data from three community-based cross sectional surveys conducted in 2007, 2010 and 2012 among 1258, 1197 and 2583 adult women, respectively were studied. In 2012, among 2583 women, 73.7% reported a preference for circumcised partners, and 87.9% knew that circumcised men could become infected with HIV. A total of 95.8% preferred to have their male children circumcised. These three proportions increased significantly during the roll-out. In 2007, the corresponding values were 64.4%, 82.9% and 80.4%, respectively. Among 2581 women having had sexual intercourse with circumcised and uncircumcised men, a majority (55.8%, 1440/2581) agreed that it was easier for a circumcised man to use a condom, 20.5% (530/2581) disagreed; and 23.07 (611/2581) did not know. However, some women incorrectly stated that they were fully (32/2579; 1.2%; 95%CI: 0.9% to 1.7%) or partially (233/2579; 9.0%; 95%CI: 8.0% to 10.2%) protected when having unprotected sex with a circumcised HIV-positive partner. This study shows that the favorable perception of women and relatively correct knowledge regarding VMMC had increased during the roll-out of VMMC. When possible, women should participate in the promotion of VMMC although further effort should be made to improve their knowledge.

## Introduction

Three randomized controlled trials published in 2005 [1] and 2007 [2,3] demonstrated that the female-to-male transmission of HIV is reduced by male circumcision. Following these trials, the World Health Organization (WHO) and the Joint United Nations Programme on HIV/AIDS (UNAIDS) recommended the use of male circumcision (MC) as an additional strategy for HIV prevention [4] in Southern and Eastern Africa. Two years following the recommendation, the roll-out of Voluntary medical MC (VMMC) started in 14 priority countries of these two African regions [5]. Such roll-outs, if successful, may yield a marked reduction of the HIV epidemic in Eastern and Southern Africa, which represents approximately 60% of new cases worldwide [6].

Women are directly involved in these roll-outs for several reasons. Firstly, male circumcision may influence sexual encounters of both men and women. Secondly, women can either encourage or discourage the roll-out by encouraging or discouraging the circumcision of their partners and their male children [7,8]. Thirdly, as HIV transmission in sub-Saharan Africa is largely heterosexual [9], the roll-out of medical MC is expected to have some effects on HIV among men [10] and thus will in turn affect the exposure of women to the virus. In this context, investigating women’s knowledge, preferences and perceptions regarding medical MC is important and should be studied in the context of the current VMMC roll-out in Southern and Eastern Africa.

To our knowledge, women’s opinions concerning MC are mostly positive. Acceptability studies carried out before implementation of roll-out in Kenya, South Africa, and Botswana, have shown that 47–79% of women were in favor of circumcision for their sexual partners and 62–89% of women were willing to circumcise their sons [11]. Another Kenyan study conducted immediately before VMMC program scale-up commenced reported a preference among women for circumcised partners[12]. However, limited community-based studies address trends of women’s opinions concerning MC over time in the context of VMMC roll-out. The Orange Farm community offers the opportunity to conduct such a study. Indeed, a VMMC roll-out, called the Bophelo Pele project, was conducted in an intense manner between 2008 and 2010.[13] At the same time independent community-based cross-sectional surveys were conducted from 2008 to 2012.[10]

The objective of this study was to elucidate the evolution of women’s preferences, acceptance and knowledge about VMMC in Orange Farm before and after the implementation of the Bophelo Project.

## Material and methods

### Study setting

The study was conducted in the township of Orange Farm, located in Gauteng province, South Africa. The township has an estimated population of 110 000 adults with a male-to-female sex ratio of 0.83. The HIV epidemic in the province is among the most severe in the world, with a prevalence estimated at 30% among women attending antenatal care in 2010 [14].

### VMMC roll-out

The Bophelo pele project (ANRS-12126) was implemented in early 2008 as a comprehensive community-based HIV prevention intervention offering free VMMC services to all men age 15 and older living in Orange Farm. It has been described elsewhere in detail [13]. In brief, project activities included community mobilization and outreach, using communication approaches aimed at both men and women, and incorporating broader HIV prevention strategies. Free medical MC was offered at the project's main center, which has been designed for low-income settings according to UNAIDS/WHO operational guidelines [15]. The roll-out led to an increase in MC prevalence among adults from 17% to 53%.[10]

### Surveys

Three independent community-based surveys, referred to here as the 2007, 2010 and 2012 surveys, were conducted in the township of Orange Farm respectively from November 2007 to April 2008, from October 2010 to June 2011 and from March to October 2012. They were designed to assess the effect of medical MC roll-out in the community. The 2007 and 2010 surveys have been described elsewhere.[10] The 2007 survey was undertaken immediately prior to the start of the roll-out of VMMC, the 2010 survey just at its end, and the 2012 survey two years after its end. In each of these surveys, a random sample of households was selected from Statistics South Africa Enumerator Area aerial photographs. All women aged 15–49 years for the 2007 and 2010 surveys and aged 18–49 years for the 2012 surveys, who had slept in the selected households the night before the study team’s visit, were eligible for inclusion. Ethical clearance for the surveys was granted by the Human Research Ethics Committee (Medical) of the University of the Witwatersrand on May 8, 2007 (protocol study number M070367). Voluntary, written informed consent was obtained, in addition to parental consent for those under 18 years. Each participant was interviewed at the study site using an anonymous structured, standardized questionnaire adapted from an instrument designed by UNAIDS [16]. These questionnaires allowed the collection of background characteristics as well as preferences, acceptance and knowledge about VMMC.

### Statistical methods

#### Factors

The following background factors were used in the analysis: age, age group, ethnic group (Sotho, Zulu, other), education level for those not at school (grade 12 completed or not completed), and ever having been married (yes, no). Sexual behavior factors were number of lifetime partners (1–2, more than 2) and having ever had sex with a circumcised man (yes, no). These factors were categorical data, with the exception of age.

The preference, knowledge and perceptions were collected using specific closed questions. When useful, some responses were grouped. Some questions were only asked in specific surveys. The information collected included the sexual preference of women, circumcision preference for their male children, the perception that circumcision increases sexual pleasure, the acceptability of circumcision for their partners and of circumcision at birth for their male newborns, support for male circumcision by their partners and family, knowledge about male circumcision, knowledge about the Bophelo Pele project, perception of what the community thought about men volunteering for circumcision and experience about male circumcision and condom use.

#### Outcomes and analysis

As recommended, the household and individual response rates were calculated [17]. For categorical data we used frequency distribution and relative frequency. Confidence intervals (CI) of proportions were calculated using the Clopper-Pearson interval by calculating quantiles from the beta distribution [18]. Age in years was analyzed with mean, median and interquartile ranges (IQR). The variations with time of dichotomous factors were analysed by estimating the linear trend of prevalence ratios (PR) and adjusted PR (aPR) using univariate and multivariate Poisson regression models. These average variations in percentage per year were calculated from aPR using the following formula: 100*(aPR-1). Multivariate analyses were controlled for age and for ethnic group, which were the only background characteristics constant with time at an individual level. Analyses were computed using the R statistical package version 3.2.5.[19] All intervals are 95% CI.

#### Populations

The study was conducted among women from the three surveys who reported at least one sexual partner. The surveys were independent. A total of 195 women (3.7% of the total) participated in more than one survey. For these women only the first participation was recorded to avoid bias due to counseling offered during each survey, which included information about male circumcision.

## Results

### Response rate

In 2007, the household response rate was 1586/1677 (94.6%), the individual response was 1401/1805 (77.6%), and the combined response rate was 73.4%. In 2010, the household response rate was 1894/1971 (96.1%), the individual response was 1376/1464 (94.0%), and the combined response rate was 90.3%. In 2012, the household response rate was 4661/5555 (83.9%), the individual response was 2811/4175 (67.3%) and the combined response rate was 56.5%.

### Background characteristics of the samples

A total of 5038 women aged 15–49 years and reporting at least one sexual partner were identified from the three surveys. The mean, median and IQR of their age in years were 29.3 (27.2; 21.2 to 36.9), 30.3 (28.3; 22.3 to 38.1) and 31.0 (29.0; 22.9 to 38.1), respectively. Their background characteristics and changes with time are described in Table 1. The Sotho and the Zulu ethnic groups accounted for approximately a third of the total number of participants. In general, the Zulus do not practice circumcision. In contrast, the Sothos practice traditional circumcision. The proportion of women ever having sex with a circumcised man reached approximately 80% in 2012 and, as expected, increased with time.

**Table 1:** Background characteristics of women from samples obtained in 2007, 2010 and 2012 in the South African township of Orange Farm.

	2007	2010	2012	Total	Average proportionate change in prevalence per year with 95%CI and p-value*
	N = 1258	N = 1197	N = 2583	N = 5038	
Age (years)					
15–27	52.2%	49.0%	46.6%	48.6%	-2.6% (-4.0% to -1.0%) p = 0.001
28–49	47.8%	51.0%	53.4%	51.4%	
Ethnic group					
Sotho	32.7%	37.3%	38.1%	36.6%	3.5% (1.4% to 5.6%) p = 0.001
Zulu	37.7%	41.7%	37.9%	38.8%	0.4% (-1.6% to 2.3%) p = 0.720
Other	29.7%	21.1%	23.9%	24.7%	-5.1% (-7.5% to -2.7%) p = 0.000
Education**					
Grade12 completed	22.3%	31.1%	32.8%	29.9%	9.5% (6.5% to 12.6%) p = 0.000
Grade12 not completed	77.7%	68.9%	67.2%	70.1%	
Ever married					
Yes	40.1%	52.1%	54.6%	50.4%	5.5% (3.8% to 7.2%) p = 0.000
No	59.9%	48.0%	45.4%	49.6%	
Number of lifetime partners					
1–2	40.5%	37.0%	34.6%	36.7%	-2.3% (-4.2 to -0.38%) p = 0.019
More than 2	59.5%	63.0%	65.4%	63.3%	
Ever had sex with a circumcised man					
Yes	72.1%	77.9%	81.2%	78.3%	2.7% (1.9% to 3.6%) p = 0.000
No	27.9%	22.1%	18.8%	31.7%	

** For those not at school

* Linear trend obtained using Poisson regression controlled for age-group (15–24, 25–34, 35–49 years) and ethnic group, when relevant.

CI: confidence interval.

### Knowledge and perception regarding male circumcision

Knowledge and perception regarding male circumcision of women sampled in the three surveys are described in Table 2. This table shows the proportion of women preferring to have sex with circumcised men increased from approximately 50% to 75%. Among 1117 women in 2012 who reported having ever had sex with both a circumcised and uncircumcised man, 909 (81.4%) reported preferring circumcised partners, 19 (1.7%) reported preferring uncircumcised partners and 189 (16.9%) did not report a preference. Table 2 shows that the vast majority of women preferred to have their male children circumcised and the corresponding proportion increased with time. Most women reported acceptance of circumcision for their partners and that family support for male circumcision increased with time. In general, this table shows the positive perception of women regarding male circumcision. In addition, as indicated by this table, most women correctly reported the need for a circumcised man to protect himself from sexually transmitted infections, including HIV, and the proportion of women reporting this opinion increased slightly with time.

**Table 2:** Knowledge and perception of women regarding male circumcision from samples obtained in 2007, 2010 and 2012 in the South African township of Orange Farm.

	2007	2010	2012	Total	Average proportionate change in prevalence per year with 95%CI and p-value*
	N = 1258	N = 1197	N = 2583	N = 5038	
**Preferences**					
Generally speaking I would prefer to have sex with a circumcised man than with an uncircumcised man					
Yes	48.3%	65.8%	73.7%	65.5%	10.1% (8.7% to 11.4%) p = 0.000
No/Same/DNK	51.7%	34.2%	26.3%	34.5%	
Most women prefer circumcised men					
Agree	64.4%	71.6%	73.7%	70.9%	3.0% (1.9% to 4.0%) p = 0.000
Disagree/DNK	35.6%	28.4%	26.3%	29.1%	
I prefer to have my male children circumcised					
Yes	80.4%	93.1%	95.8%	91.2%	4.0% (3.5% to 4.5%) p = 0.000
No	19.6%	6.9%	4.2%	8.8%	
**Perception**					
Circumcision increases pleasure during sex					
Agree	41.5%	47.8%	59.6%	52.3%	8.4% (6.7% to 10.1%) p = 0.000
Disagree	15.1%	12.9%	14.7%	14.4%	-0.6% (-4.2% to 3.2%) p = 0.770
DNK	43.4%	39.3%	25.7%	33.4%	-10.1% (-11.9% to -8.3%) p = 0.000
**Acceptability**					
If I have an uncircumcised partner I would accept that he undergo circumcision					
Yes	89.7%	95.7%	93.3%	93.0%	0.9% (0.5% to 1.3%) p = 0.000
No	10.3%	4.3%	6.7%		
If circumcision was offered free at birth, I would have my male children circumcised					
Yes	58.8%	68.2%	74.0%	68.8%	4.9% (3.8% to 6.0%) p = 0.000
No	41.2%	31.8%	26.0%	31.2%	
**Family and partner support**					
My partner supports circumcision					
Agree	57.9%	72.4%	75.3%	70.2%	6.1% (5.0% to 7.2%) p = 0.000
Disagree/DNK	42.1%	27.6%	24.7%	29.8%	
My family supports circumcision					
Agree	56.6%	73.4%	80.0%	72.6%	8.1% (7.0% to 9.2%) p = 0.000
Disagree/DNK	43.4%	26.6%	20.0%	27.4%	
**Knowledge towards MC**					
Circumcised men need to use condoms to protect them from STIs and HIV					
Agree	82.9%	86.0%	87.9%	86.2%	1.4% (0.8% to 2.0%) p = 0.000
Disagree/DNK	17.1%	14.0%	12.1%	13.8%	

* Linear trend obtained using Poisson regression controlled for age-group (15–24, 25–34, 35–49 years) and ethnic group.

CI: confidence interval.

STI: sexually transmitted infection

DNK: Don't know

The opinions of women in 2010 concerning men volunteering for circumcision are reported in Table 3. In total, "Protecting his health", "Being responsible” and "Becoming a man" represented 79.3% (95%CI: 77.0% to 81.6%) of the opinions of women regarding reasons for men to volunteer for such a procedure. The negative opinions ("He is foolish to get the procedure", "He is losing his culture", "He is planning to have sex just anyhow" and "He is cowardly") of women about a man who volunteered for circumcision decreased from 13.7% (172/1255) in 2007 to 4.3% (51/1196) in 2010 with an average adjusted variation in percentage per year of (-31.4%; -37.9% to -24.6%; p<0.001).

**Table 3:** Main opinions of women in 2010 concerning men volunteering for circumcision in the South African township of Orange Farm. The question was "What do people in your community think about men volunteering for MC?", and only the first answers were reported.

	% (Number of responses; 95% confidence interval) N = 1196
**Positive opinion**	
He is protecting his health	35.2% (421; 32.5% to 37.9%)
He is now considered as a man	26.0% (311; 23.6% to 28.5%)
He is being responsible	18.1% (217; 16.0% to 20.4%)
He is intelligent	1.8% (21; 1.1% to 2.6%)
He is taking care of his partner	1.3% (16; 0.8% to 2.1%)
He is brave to get the procedure	1.0% (12; 0.5% to 1.7%)
**Negative opinion**	
He is planning to have sex just anyhow	1.5% (18; 0.9% to 2.3%)
He is losing his culture	1.3% (16; 0.8% to 2.1%)
He is cowardly	0.9% (11; 0.5% to 1.6%)
He is foolish to get the procedure	0.5% (6; 0.2% to 1.0%)
**Other opinion (either negative or positive)**	12.3% (147; 10.5% to 14.2%)

### Additional information collected in 2012

In 2012 additional information was collected from the 2583 women included in the 2012 survey. The best age for MC reported by these women had a bimodal distribution, with a first peak at the age of 0 year (13.2% of the sample), and a second peak between 15 and 18 years of age (48.6% of the sample). In women not reporting birth as best the time for MC, the reported best age was on average 13.8 years with a median (IQR) of 15 years (11 to 18). Regarding knowledge of the project, 59.9% (58.0% to 61.8%) of women recognized the name of the Bophelo Pele project and its aims. Among these 1548 women, 95.1% (93.9% to 96.1%) thought it was good for the Orange Farm community. When shown drawings of an uncircumcised penis, a circumcised penis and a penis with a foreskin partially covering the glans, 84.8% (83.4% to 86.2%) of women could correctly recognize the circumcised penis. Some women stated that they were fully 1.2%; (0.9% to 1.7%) or partially 9.0%; (8.0% to 10.2%) protected when having unprotected sex with a circumcised HIV-positive partner. When asked about circumcised men, 1.8% (1.4% to 2.4%) of women stated that such men were fully protected against HIV, whereas 49.3% (47.4% to 51.2%) asserted that these men had a lower HIV risk than uncircumcised men. Among women having had sex with circumcised and uncircumcised men, a majority, 55.8% (53.9% to 57.7%), agreed that it is easier for a circumcised man to use a condom, 20.5% (19.0% to 22.1%) disagreed with this opinion and 23.7% (22.1% to 25.3%) did not know. Lastly, 12.7% (11.4% to 14.0%) of women reported that at least one male partner in the past year refused to use condoms, claiming that as a circumcised man he did not need condoms in order to prevent possible transmission of HIV.

## Discussion

### Main results

By conducting large community-based cross sectional surveys among random samples of women in the township of Orange Farm in South Africa before and after the roll-out of VMMC, we found that women’s favorable opinion of male circumcision was already high before the roll-out and increased with time. Women had, on average, good knowledge about MC and they preferred circumcised sexual partners over uncircumcised men. Women’s favorable opinion of male circumcision increased with time. However, we found that approximately 10% of women incorrectly seemed to think that male circumcision reduced the risk of male-to-female transmission of HIV.

### Limitations

To our knowledge, this is the first time that temporal changes in women’s knowledge and opinions regarding circumcision have been described. However, our study has some limitations. Firstly, when shown drawings of an uncircumcised penis, not all women could correctly recognize the circumcised penis, which can introduce erroneous results. Secondly, our study neither explores personal motivators nor the gap between hypothetical sexual preference-acceptance and concrete practice. Thirdly, the 15–17 years’ age-group was not sampled in 2012. These women account for 6.6% of the women sampled in 2007 and 2010. We recalculated Table 2 by including only the participants aged 18–49 years. The results are shown in supplemental information (S1 Table). The differences between Table 2 and S1 Table are minor. These elements, together with the fact that variations with time were adjusted in particular for age group, indicated that the corresponding bias is small. Lastly, as it has not been demonstrated that HIV incidence is lower in women who have a circumcised partner, we cannot conclude that women's health will benefit from choosing circumcised partners.

### Interpretation

This study is observational. As a consequence, no causal association between the roll-out of VMMC in Orange Farm and observed variation with time can be drawn from the results. However, the roll-out likely played a role in the change with time of the knowledge and perception that women had regarding MC. Some other factors such as information regarding MC on television, radio and newspapers, or the information reported by men to women, or even by children who were exposed to talks at school, could also have played a role. Indeed, these campaigns started in South Africa in 2010. It is also possible that the positive view of women regarding MC and its increased prevalence with time may be associated with the increased understanding by women that circumcised men are less likely to become infected with HIV, as it has been reported in Kenya [20].

### Comparison with other results

Our results are consistent with other studies devoted to women’s perceptions of MC. A community-based survey conducted in Kenya has shown that preference for circumcised men was associated with improvement in sexual pleasure, and perceptions of impact on condom utilization [21]. Similarly, a study conducted in Uganda reported improved sexual satisfaction among women after their male partner was circumcised [22].

We were surprised to observe that a majority of women were in favor of MC for their children in the survey conducted before the beginning of the roll-out, at a time when MC prevalence was low in this community. This is consistent with an acceptability review study conducted in 9 countries in 2007. In Kenya, as shown by a community-based survey, factors affecting opinion included a belief that circumcision was not part of the local culture, the perception of a long healing period following the procedure, the lack of a specific motivation to seek out services, and the general fear of pain associated with the circumcision procedure and healing process [23]. Finally, misinformation regarding the association of MC and HIV risk has been revealed in qualitative studies conducted in Tanzania [24] and South Africa [25].

## Conclusions

Women’s favorable opinion of male circumcision found in this study and in other studies are encouraging for the ongoing roll-out of VMMC in Southern and Eastern Africa. These results confirm that women should be involved in the efforts to recruit men for VMMC, as female perception of circumcision may be significantly influential in a man’s decision to undergo MC [7,17,25]. This involvement, however, is not necessarily feasible throughout Eastern and Southern Africa due to cultural resistance, in particular among health providers, such as described in Malawi [26]. Female involvement and their positive influence upon MC prevalence could be increased by encouraging discussions about MC within families and households and by conducting talks with school children of appropriate ages.

## Supporting information

S1 Table Knowledge and perception of women aged 18 to 49 years regarding male circumcision from the samples obtained in 2007, 2010 and 2012 in the South African township of Orange Farm. (PDF)

## References

[1] Auvert B, Taljaard D, Lagarde E, Sobngwi-Tambekou J, Sitta R, Puren A. Randomized, controlled intervention trial of male circumcision for reduction of HIV infection risk: the ANRS 1265 Trial. PLoS Med. 2005;2: e298 10.1371/journal.pmed.0020298 16231970 PMC1262556

[2] Gray RH, Kigozi G, Serwadda D, Makumbi F, Watya S, Nalugoda F, et al Male circumcision for HIV prevention in men in Rakai, Uganda: a randomised trial. Lancet. 2007;369: 657–66. 10.1016/S0140-6736(07)60313-4 17321311

[3] Bailey RC, Moses S, Parker CB, Agot K, Maclean I, Krieger JN, et al Male circumcision for HIV prevention in young men in Kisumu, Kenya: a randomised controlled trial. Lancet. 2007;369: 643–56. 10.1016/S0140-6736(07)60312-2 17321310

[4] WHO-UNAIDS. Technical Consultation on Male Circumcision and HIV Prevention: Research Implications for Policy and Programming. New Data Male Circumcision HIV Prev Policy Programme Implic. 2007; Available: http://www.who.int/hiv/mediacentre/MCrecommendations_en.pdf

[5] Reed JB, Njeuhmeli E, Thomas AG, Bacon MC, Bailey R, Cherutich P, et al Voluntary medical male circumcision: an HIV prevention priority for PEPFAR. J Acquir Immune Defic Syndr 1999. 2012;60 Suppl 3: S88–95. 10.1097/QAI.0b013e31825cac4e PMC3663585 22797745

[6] UNAIDS. UNAIDS Report on the global AIDS epidemic. Geneva; 2012.

[7] Lanham M, L’engle KL, Loolpapit M, Oguma IO. Women’s roles in voluntary medical male circumcision in Nyanza Province, Kenya. PloS One. 2012;7: e44825 10.1371/journal.pone.0044825 23028634 PMC3446991

[8] Lissouba P, Taljaard D, Rech D, Dermaux-Msimang V, Legeai C, Lewis D, et al Adult male circumcision as an intervention against HIV: An operational study of uptake in a South African community (ANRS 12126). BMC Infect Dis. 2011;11: 253 10.1186/1471-2334-11-253 21943076 PMC3192707

[9] De Cock KM, Jaffe HW, Curran JW. The evolving epidemiology of HIV/AIDS. AIDS Lond Engl. 2012;26: 1205–1213. 10.1097/QAD.0b013e328354622a 22706007

[10] Auvert B, Taljaard D, Rech D, Lissouba P, Singh B, Bouscaillou J, et al Association of the ANRS-12126 male circumcision project with HIV levels among men in a South African township: evaluation of effectiveness using cross-sectional surveys. PLoS Med. 2013;10: e1001509 10.1371/journal.pmed.1001509 24019763 PMC3760784

[11] Westercamp N, Bailey RC. Acceptability of Male Circumcision for Prevention of HIV/AIDS in Sub-Saharan Africa: A Review. AIDS Behav. 2006; 10.1007/s10461-006-9169-4 PMC1847541 17053855

[12] Westercamp M, Agot KE, Ndinya-Achola J, Bailey RC. Circumcision preference among women and uncircumcised men prior to scale-up of male circumcision for HIV prevention in Kisumu, Kenya. AIDS Care. 2012;24: 157–166. 10.1080/09540121.2011.597944 21854351 PMC3682798

[13] Lissouba P, Taljaard D, Rech D, Doyle S, Shabangu D, Nhlapo C, et al A model for the roll-out of comprehensive adult male circumcision services in African low-income settings of high HIV incidence: the ANRS 12126 Bophelo Pele Project. PLoS Med. 2010;7: e1000309 10.1371/journal.pmed.1000309 20652013 PMC2907271

[14] National Department of Health P. The 2010 National antenatal sentinel HIV and syphilis prevalence survey in South Africa. 2011 pp. 50–53.

[15] UNAIDS-WHO. Operational guidance for scaling up male circumcision services for HIV prevention [Internet]. 2008. Available: http://www.malecircumcision.org/programs/documents/MC_OpGuideFINAL_web.pdf

[16] UNAIDS. Looking deeper into the HIV epidemic: A questionnaire for tracing sexual networks, in Best practice collection, Key materiall 98/27. UNAIDS Geneva 1998; 1–24.

[17] Designing Household Survey Samples: Practical Guidelines. Studies in Methods. Series F No.98. Designing Household Survey Samples: Practical Guidelines. Logo. United Nations. New York, 2005. Page 113.

[18] Clopper C., Pearson E. S. The use of confidence or fiducial limits illustrated in the case of the binomial. Biometrika. 1934;26: 404–413.

[19] Team RDC. R: A language and environment for statistical computing. R Found Stat Comput Vienna Austria 2005;

[20] Westercamp M, Bailey RC, Bukusi EA, Montandon M, Kwena Z, Cohen CR. Male circumcision in the general population of Kisumu, Kenya: beliefs about protection, risk behaviors, HIV, and STIs. PloS One. 2010;5: e15552 10.1371/journal.pone.0015552 21179493 PMC3002946

[21] Westercamp M, Agot KE, Ndinya-Achola J, Bailey RC. Circumcision preference among women and uncircumcised men prior to scale-up of male circumcision for HIV prevention in Kisumu, Kenya. AIDS Care. 2012;24: 157–166. 10.1080/09540121.2011.597944 21854351 PMC3682798

[22] Kigozi G, Lukabwe I, Kagaayi J, Wawer MJ, Nantume B, Kigozi G, et al Sexual satisfaction of women partners of circumcised men in a randomized trial of male circumcision in Rakai, Uganda. BJU Int. 2009;104: 1698–1701. 10.1111/j.1464-410X.2009.08683.x 19522862

[23] Westercamp M, Agot KE, Ndinya-Achola J, Bailey RC. Circumcision preference among women and uncircumcised men prior to scale-up of male circumcision for HIV prevention in Kisumu, Kenya. AIDS Care. 2012;24: 157–166. 10.1080/09540121.2011.597944 21854351 PMC3682798

[24] Layer EH, Beckham SW, Mgeni L, Shembilu C, Momburi RB, Kennedy CE. “After my husband’s circumcision, I know that I am safe from diseases”: women’s attitudes and risk perceptions towards male circumcision in Iringa, Tanzania. PloS One. 2013;8: e74391 10.1371/journal.pone.0074391 24009771 PMC3756960

[25] Mantell JE, Smit JA, Saffitz JL, Milford C, Mosery N, Mabude Z, et al Medical male circumcision and HIV risk: perceptions of women in a higher learning institution in KwaZulu-Natal, South Africa. Sex Health. 2013;10: 112–118. 10.1071/SH12067 23448912 PMC3963517

[26] Umar E, Mandalazi P, Jere D, Muula A. Should female health providers be involved in medical male circumcision? Narratives of newly circumcised men in Malawi. Malawi Med J J Med Assoc Malawi. 2013;25: 72–77. PMC3859992 24358423

