# Supplementary material for: Women’s knowledge and perception of male circumcision before and after its roll-out in the South African township of Orange Farm from community-based cross-sectional surveys
Source: PLoS One. 2017 Mar 24;12(3):e0173595. doi: 10.1371/journal.pone.0173595 (PMC5365100; doi:10.1371/journal.pone.0173595)
Supplement: S1 Table — (PDF) [file pone.0173595.s001.pdf]

S1 Table. Knowledge and perception of women of ages 18 to 49 years regarding male circumcision from the samples obtained in 2007, 2010 and 2012 in the South African township of Orange Farm.

	2007<br>N= 1160	2010<br>N=1133	2012<br>N=2583	Total<br>N=4876	Average proportionate change in prevalence per year with 95%CI and p-value*
**Preferences**					
Generally speaking would you prefer to have sex with a circumcised man rather than with an uncircumcised man					
Yes	49.8%	65.7%	73.7%	66.1%	9.3% (8.0% to 10.7%) p=0.000
No/Same/DNK	50.2%	34.3%	26.3%	33.9%	
Most women prefer circumcised men					
Agree	66.1%	71.0%	73.7%	71.3%	2.4% (1.4% to 3.5%) p=0.000
Disagree/DNK	33.9%	29.0%	26.3%	28.7%	
I prefer to have my male children circumcised					
Yes	81.8%	93.1%	95.8%	91.8%	3.6% (3.1% to 4.1%) p=0.000
No	18.2%	6.9%	4.2%	8.2%	
**Perception**					
Circumcision increases pleasure during sex					
Agree	43.5%	48.4%	59.6%	53.2%	7.4% (5.8% to 9.2%) p=0.000
Disagree	14.7%	12.8%	14.7%	14.2%	0.1% (-3.7% to 4.1%) p=0.950
DNK	38.8%	38.8%	25.7%	32.6%	-9.7% (-11.6% to -7.8%) p=0.000
**Acceptability**					
If I have an uncircumcised partner I would accept that he undergoes circumcision					
Yes	90.8%	95.9%	93.3%	93.3%	0.6% (0.2% to 1.1%) p=0.003
No	9.2%	4.1%	6.7%	6.7%	
If circumcision was offered free at birth, I would have my male children circumcised					
Yes	61.5%	69.1%	74.0%	69.9%	4.1% (3.0% to 5.2%) p=0.000
No	38.5%	30.9%	26.0%	30.1%	
**Family and partner support**					
My partner supports circumcision					
Agree	58.7%	72.2%	75.3%	70.6%	5.8% (4.7% to 6.9%) p=0.000
Disagree/DNK	41.3%	27.8%	24.7%	29.4%	
My family supports circumcision					
Agree	57.2%	74.1%	80.0%	73.2%	7.9% (6.8% to 9.0%) p=0.000
Disagree/DNK	42.8%	25.9%	20.0%	26.8%	
**Knowledge towards MC**					
Circumcised men need to use condoms to protect them from STIs and HIV					
Agree	82.9%	86.4%	87.9%	86.4%	1.4% (0.7% to 2.0%) p=0.000
Disagree/DNK	17.1%	13.6%	12.1%	13.6%	

* Linear trend obtained using Poisson regression controlled for age-group (18-24, 25-34, 35-49 years) and ethnic group.
CI: confidence interval.
STI: sexually transmitted infection
DNK: Don't know
